# Copy number variations and protein expression of Cyclin E1 and Epithelial cell transforming sequence 2 genes predict the chemotherapeutic response in patients with serous ovarian carcinoma

**DOI:** 10.12669/pjms.39.3.7304

**Published:** 2023

**Authors:** Rahat Sarfraz, Atika Masood, Saima Zaki, Amira Shami, Saba Khaliq, Nadia Naseem

**Affiliations:** 1 Rahat Sarfraz, Department of Morbid Anatomy and Histopathology, University of Health Sciences, Lahore, Pakistan; 2 Atika Masood, Department of Morbid Anatomy and Histopathology, University of Health Sciences, Lahore, Pakistan; 3 Saima Zaki, Associate Professor, Department of Obstetrics & Gynaecology, Jinnah Hospital, Lahore, Pakistan; 4 Amira Shami, Consultant Oncologist, Department of Radiation Oncology, Institute of Nuclear Medicine and Oncology, Lahore, Pakistan; 5 Saba Khaliq, Associate Professor, Department of Physiology & Cell Biology, University of Health Sciences, Lahore, Pakistan; 6 Nadia Naseem, Professor & Head, Department of Morbid Anatomy and Histopathology, University of Health Sciences, Lahore, Pakistan

**Keywords:** Chemotherapy response, Copy number variants, Cyclin E1 gene, Epithelial cell transforming sequence 2 gene, Serous Ovarian Carcinoma

## Abstract

**Background & Objectives:**

Serous ovarian carcinoma (SOC) is characterized by extreme genomic instability, chromosomal rearrangements and copy number variations (CNVs) leading to the development of early metastasis and chemo-resistance. The present study was designed to observe the role of CNVs of Cyclin E1 (CCNE1) and Epithelial cell transforming sequence- 2 *(ECT2)* genes and their encoded proteins in predicting the chemotherapeutic response in SOC patients**.**

**Methods:**

This observational analytical study was conducted at University of Health Sciences, Lahore, Pakistan from December 2019 till June 2022.The study included twenty-five SOC patients with resectable ovarian tumors and twenty-five control subjects. The patients were followed-up for six months for their response to chemotherapy. The CNVs in *CCNE1* and *ECT-2* genes were determined by real time PCR while serum levels of encoded proteins were determined in controls and cases, before and after six months of treatment, through ELISA. The response to chemotherapy was categorized as sensitive or resistant based on serum CA-125 levels and radiological scans.

**Results:**

The copy number variations in *CCNE1* and *ECT2 genes* showed association with the clinic-pathological characteristics and chemotherapy response. Statistically significant difference was found between the mean pre-chemotherapy protein levels of *CCNE1* in cases than controls (p-value <0.001) and between the mean pre and post-chemotherapy protein levels of *CCNE1 and ECT2 (*p-value <0.001) in SOC patients.

**Conclusion:**

The copy number variations of *CCNE1* and *ECT2 genes* and their protein expression are positively associated with chemotherapeutic response in SOC patients.

## INTRODUCTION

Ovarian cancer (OC) is the most common cause of death in females diagnosed with gynecological cancers.^1^ According to 2020 GLOBOCAN data, 314,000 women were diagnosed with OC leading to 207,000 deaths due to the disease, making it the eighth common cause in terms of both cancer incidence and mortality worldwide.^2^ Annual cancer registry statistics of Shaukat Khanum Memorial Cancer Hospital & Research Centre, 2021 reported OC as the 3^rd^ most common malignancy among adult females.^3^

Epithelial ovarian cancer (EOC) is the most common and lethal among all kinds of OC.^4^,^5^ Serous ovarian carcinoma (SOC) makes up the most common histological subtype of EOCs and is divided into High grade serous ovarian carcinoma (HGSOC) and Low-grade serous ovarian carcinoma (LGSOC).^6^,^7^ Extensive cytoreductive surgery combined with platinum-based chemotherapy is currently the standard treatment for most of the patients without consideration of individual prognostic and predictive biomarkers. First-line platinum chemotherapy is highly effective but unfortunately more than 80% of patients relapse and become platinum-resistant. The molecular mechanisms responsible for chemo-resistance are still unclear.^5^

HGSOC have a high frequency of copy number variations (CNVs) and low mutation rate. CNVs affect a large fraction of the genome resulting in the amplification or loss of several genes leading to genomic instability.^6^ In the process of carcinogenesis, CNVs are involved in the growth, progression and treatment response.^8^ There are gains and losses in copy numbers. Based on their location, content and size they result in an increase or decrease in gene expression directly or indirectly through position effect, by unmasking recessive mutations or by altering communication between alleles.^9^

*Cyclin E1* (*CCNE1*) gene is a master regulator of progression of cell cycle through G1/S phase. Overexpression of *CCNE1* results in dysregulation of G1-S checkpoint and has a critical role in the development of malignancy including epithelial ovarian carcinoma.^10^ Epithelial cell transforming sequence 2 (*ECT2*), a guanine nucleotide exchange factor is a proto-oncogene. It has the ability to transform NIH/3T3 fibroblasts into malignant cells. High expression of *ECT2* is associated with unfavorable prognosis in different types of cancers.^11^

As molecular targets relating the treatment response are unclear so it is necessary to get a more comprehensive insight into the pathogenesis of the disease for the development of new targeted drugs. To date, no data in Pakistan is available regarding role of CNVs in SOC patients. Keeping in view the documented role of CNVs in tumor biology and heterogeneous treatment response of these tumors, the present study was designed to determine the role of CNVs of *CCNE1 and ECT2*genes and their protein expression as possible predictors of chemotherapy response in SOC patients. This might help in providing prognostic and therapeutic information to treating oncologist minimizing chances of chemo-resistance and treatment failure. In addition, it may reduce the morbidity and mortality with an improved progression free and overall survival of patients by predictive stratification and tailoring individual treatment for patients.

## METHODS

This was an observational analytical multi-centered study conducted at the University of Health Sciences (UHS), Lahore, Institute of Nuclear Medicine and Oncology (INMOL), Jinnah Hospital and Mayo hospital. The research work was carried out in the Department of Morbid Anatomy and Histopathology and Department of Physiology & Cell Biology, UHS, Lahore from December 2019 till June 2022. After approval from Ethical Review Board of UHS vide letter no (UHS/ REG-19/ERC/4204, dated: 19 December, 2019). A written informed consent was taken from each patient recruited in the study as per Helsinki declaration.^12^

The study comprised of twenty-five consenting female patients diagnosed for the first time with resectable SOC and the same number of control subjects.

***The inclusion criteria*** for cases was, adult female patients (18-70 years) diagnosed for the first time with SOC, of any grade and any stage, Females undergoing any one of the following types of unilateral or bilateral surgery for SOC. Hysterectomy with salpingo-oophorectomy, salpingo-oophorectomy, oophorectomy, subtotal resection or removal of tumour fragments either by laparotomy or laproscopically. Inclusion criteria for controls was adult female subjects (18-70 years) undergoing gynecological surgery for reasons other than SOC or any other gynaecological or non gynaecological malignancy and removal of normal ovary as part of procedure. Subjects with no family history of breast and/or ovarian cancer and/or colonic cancer were included.

***Exclusion criteria*** was patients with history of any autoimmune or chronic inflammatory disorder or malignancy, patients on follow up /treatment or recurrence of tumor, lost to follow up cases within six months after chemotherapy. Tumors with benign and borderline serous morphology or any other histological type of EOC on microscopy were also excluded.

Relevant present, past medical history and family history was taken on predesigned proformas for both cases and controls. After having detailed clinicopathological, radiological assessment and pretreatment CA-125 levels of cases, surgery followed by chemotherapy was planned.

Five milliliters of venous sample was drawn by using aseptic technique from control subjects on enrollment and cases before and six months after treatment. Within two hours of collection, the sample was centrifuged at 4000 rpm for ten minutes at 4°C to separate serum. The serum was aliquoted, labeled and stored at -80°C till it was analyzed for protein expression by ELISA.

A representative portion (0.5-1gm) of the excised fresh malignant ovarian tissue from cases and healthy ovarian tissue from control subject was snapped frozen in liquid nitrogen immediately after surgical resection and stored at -80°C for CNV analysis by RT-PCR. Rest of the ovarian tissue was kept in formalin for histopathology reporting.

Grossing of the surgical specimens was done according to the College of American Pathologists (CAP) protocol.^13^ Representative sections were taken for routine tissue processing and Hematoxylin and Eosin staining. Histopathological reporting of the tumor confirming serous carcinoma histology was done by two senior consultant histopathologists according to CAP guidelines^13^ (Fig.1 a,b,c,d). Updated International Federation of Gynecological and Obstetrics **(**FIGO)^14^ staging was done for each enrolled patient. Pre-surgery serum CA-125 levels were determined through ELISA.

**Fig.1(a):**
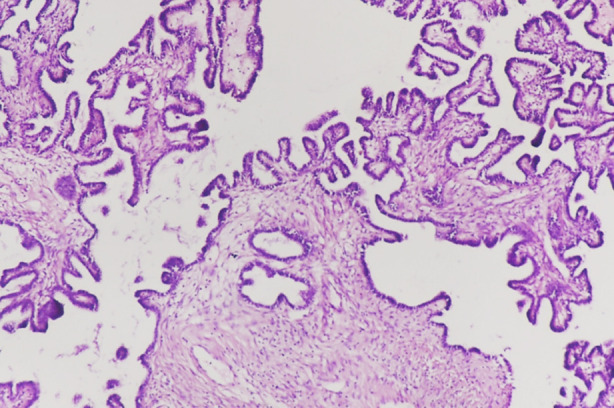
Photomicrograph: Low grade serous carcinoma: Fine papillae lined with small cells showing minimal nuclear atypia. Stromal invasion present (20x).

**Fig.1(b):**
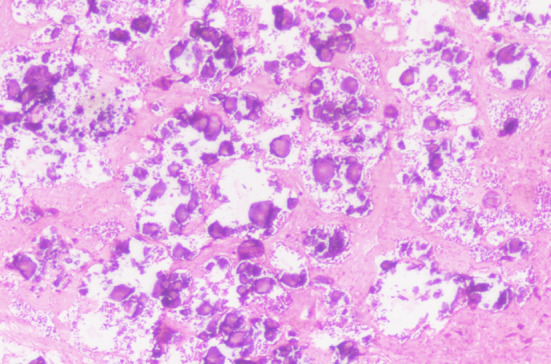
Photomicrograph: Low grade serous carcinoma: Psammomatous area (20x).

**Fig.1(c):**
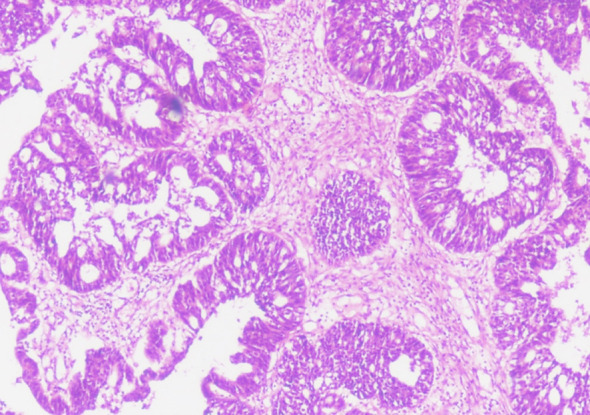
Photomicrograph: High grade serous carcinoma: Solid, cribriform growth pattern. Cells showing high grade nuclear atypia with pleomorphism (20x).

**Fig.1(d):**
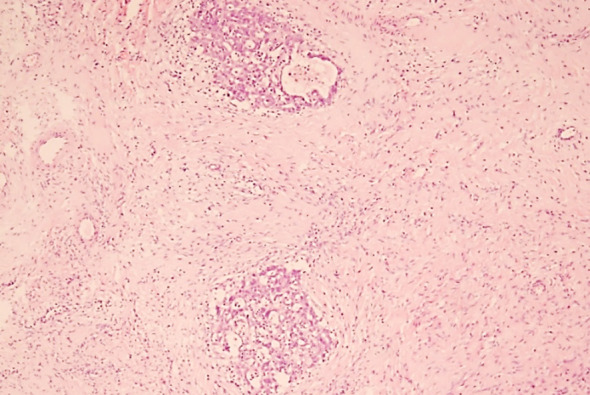
Photomicrograph: High grade serous carcinoma: Nests of tumor invading myometrium (20x).

### DNA extraction and Detection of CNVs:

Each frozen tissue sample was grounded into small pieces in liquid nitrogen by using a tissue homogenizer. The DNA extraction was done by DNA extraction Thermo Fischer Lithuania kit (cat #K0881, #K0882). DNA samples were used to experimentally validate putative CNVs at genomic regions using gene specific primers on CFX96 RT-PCR (BIORAD, USA). Primer sequences for CNVs were taken from a previous study^15^ and designed using NCBI, Primer blast. *HBB* was used as an endogenous reference gene (Table-I). All assays were performed in triplicate.

**Table-I:** Primer sequences of CNVs for qRT-PCR.

Gene	CNVs Primers	Bp-sizes	Annealing temperature
*HBB*	Forward: CACCAACTTCATCCACGTTCA Reverse:GTGCATCTGACTCCTGAGGAGAA	73 bp	58º C
*CCNEI*	Forward: CTGGGCAAATAGAGAGGAAGTC Reverse: CATGAAGCGAACAGGAAGACTC	166 bp	58^º^ C
*ECT2*	Forward: CCTTCCATGTTTCCCCTCCC Reverse: GGACTGGAGTCAAGGGCTTC	97 bp	60^º^C

### Protein Expression Analysis:

Determination of protein levels of *CCNE1 and ECT2* were done on serum samples from cases (before and six months after chemotherapy) and control subjects on recruitment through ELISA. Human G1/S-specific cyclin-E1 ELISA Kit (Cat No: E5313 Hu; Bioassay Technology Laboratory, Korain Biotech Co., Ltd.) and Human Protein ECT2 ELISA Kit (Cat No: E7177Hu; Bioassay Technology Laboratory, Korain Biotech Co., Ltd) was used for CCNEI and ECT2 according to the manufacturer’s protocol.

### Chemotherapy response:

After surgery and confirmation of diagnosis the patients were enrolled for standard first-line chemotherapy comprising of a Taxane with the addition of Carboplatin (Inj. Paclitaxel intravenous with carboplatin in combination at one time, three weekly for six cycles with dose of paclitaxel ranging from 135 to 175 mg/m^2^ and injection carboplatin in dose of 300 mg/m^2^). During chemotherapy, patients’ baseline laboratory profile including complete blood count, renal and liver function tests were monitored regularly. Each patient was followed-up for 6-months after the completion of chemotherapy in order to observe the treatment response. The response was categorized as sensitive or resistant based on CA-125 levels and radiological scans according to National comprehensive cancer network guidelines.^16^

### Statistical Analysis:

The data was entered and analyzed using Statistical Package for Social Sciences (SPSS) version 26. Mean ± SD was given for Numerical variables (Age, CA-125 levels, protein levels of *CCNE1* and *ECT2*). Frequencies and percentages were given for categorical variables (CNVs of *CCNE1* and *ECT2*, Grade and Stage of tumor). A p-value of <0.05 was considered statistically significant for all purposes. Logistic regression analysis was applied to find out the association between CNVs and chemotherapy response. Mann-Whitney test was applied to compare pre-chemotherapy mean protein levels in cases and controls. Wilcoxon-signed rank test was applied to find the difference between pre and post chemotherapy CA-125 levels in cases and also in chemo-sensitive and resistant group. The test was also applied to find out difference between mean pre- and post-chemotherapy protein levels in cases.

## RESULTS

In the present study, twenty five female subjects were enrolled in cases and control group each. The descriptive characteristics of cases and control group are given in Table-II a. In control group the mean age was 45.60 ± 6.69 years (Age range: 38-65). All were married. Menstrual regularity was present in sixteen (64.0%) subjects. Twenty three (92.0%) were pre-menopausal. The history of oral contraceptive use was given by 6 (24%) subjects.

**Table-II-a:** Descriptive characteristics of cases and controls.

Descriptive characteristics	Cases n=25				Controls n=25		

	No. (%)		Mean ± SD		No. (%)		Mean ± SD
Mean age (years)	---		47.160 ± 12.239		---		45.6 ± 6.7
***Marital status***							
Married	24 (96.0%)		---		25 (100.0%)		---
Unmarried	1(4.0%)	---		Nil		---	
Mean age at menarche (years)	---	13.680 ± 1.464		---		12.3 ± 0.80	
***Menstrual regularity***							
Present	18 (72.0%)	---		16 (64.0%)		---	
Absent	7 (28.0%)	---		9 (36.0%)		---	
***Pre-menopausal***							
Yes	9 (36.0%)	---		23 (92.0%)		---	
No	16 (64.0%)	---		2 (8.0%)		---	
***Parity (n=)***							
Multiparous	21 (84.0%)	---		24 (96.0%)		---	
Nulliparous	4 (16.0%)	---		1 (4.0%)		---	
***Oral contraceptive use***							
Yes	1(4%)			6(24.0%)		---	
No	24(96%)			19(76.0%)		---	
Duration of illness (months)	---	6.02 ± 7.15				7.58±5.044	
***Histological grade***							
High	23 (92.0%)	---					
Low	2 (8.0%)	---					
***FIGO stage***							
I	5 (20.0%)	---					
II	3 (12.0%)	---					
III	10 (40.0%)	---					
IV	7 (28.0%)	---					
***Chemotherapy response***							
Sensitive	20 (80.0%)	---					
Resistant	5 (20.0%)	---					

Statistical test applied: For numerical variables: Mean ± SD, For qualitative variables: Frequency and percentage.

The mean age of the 25 cases was 47.16 ± 12.24 years (Age range 18-70) years. Twenty one (84%) were married. The menstrual regularity was present in 18 (72.0%) cases. Sixteen (64%) cases were pre-menopausal. The history of oral contraceptive use was given by one patient The mean duration of disease before diagnosis was 6.02±7.15 months (Table-II a).

Past medical history was insignificant both in cases and controls. Family history of any type of malignancy specifying breast, ovary and colon was taken but not sufficient data was given by participants either due to lack of information or lack of education about the disease.

On histopathological analysis, 23 (92.0%) cases were reported as HGSOC while 2 (8.0%) were reported as LGSOC. According to the FIGO staging system, 05 (20.0%), 03 (12.0%), 10 (40.0%), and 07 (28.0%) subjects had FIGO Stage-I, II, III, and IV, respectively. Six months after chemotherapy, 20 (80.0%) cases were categorized as chemo-sensitive while 05 (20.0%) were chemo-resistant (Table-II a). This was based on rising levels of CA-125 and recurrence of disease with metastatic deposits in para-aortic lymph nodes, lungs, bones and liver on radiological assessment.

Four (80%) cases in chemo-resistant group were in FIGO Stage-IV, while one case was in Stage-III at the time of enrolment. The mean age of the cases in chemo-resistant group was high (54.60 ± 14.673) years compared to chemo-sensitive group (45.30 ± 11.21) years. The mean duration of illness was 6.40 ± 9.84 months in chemo-resistant group and was 5.92 ± 6.65 months in chemo-sensitive group. The presenting complaints in chemo-sensitive and chemo-resistant cases are given in (Table-II b)

**Table-II-b:** Presenting complaints of cases in chemo-sensitive and chemo-resistant group.

Chemotherapy Response		Chemo-Sensitive	Chemo-Resistant
No. of patients		n=20	n=5
Urinary symptoms	Present	11	3
	Absent	9	2
Heavy menstrual bleeding	Present	2	0
	Absent	18	5
Post-menopausal bleeding	Present	1	0
	Absent	19	5
Weight loss	Present	7	3
	Absent	13	2
Abdominal mass	Present	1	1
	Absent	19	4
Abdominal distention	Present	14	5
	Absent	6	0
Abdominal Pain	Present	19	5
	Absent	1	0

Statistical test applied: Frequency.

On analyzing the mean levels of CA-125 (U/mL) in pre and post-chemotherapy patients, it was found that the mean pre-chemotherapy CA 125 levels (1452.02±2154.97) U/ml were markedly reduced (379.31±808.65) U/ml after chemotherapy (p<0.001). In chemo-sensitive cases, the mean CA-125 levels (U/mL) before and after chemotherapy was 945.28 ± 1389.37 and 50.12 ± 73.71 respectively (p<0.001). The mean CA-125 levels (U/mL) before and after the chemotherapy in the chemo-resistant cases was 3479.00 ± 3503.50 and 1695.06 ± 1091.75 (U/mL), respectively (p<0.225). This shows that decrease in levels were statistically significant in chemo-sensitive than chemo-resistant cases. Seventeen cases (68%) showed CNVs of *CCNE1* and 23 cases (92%) showed *ECT2* CNVs. Both copy number gain (CNG) and copy number loss (CNL) were observed (Fig.2(a)).

**Fig.2(a):**
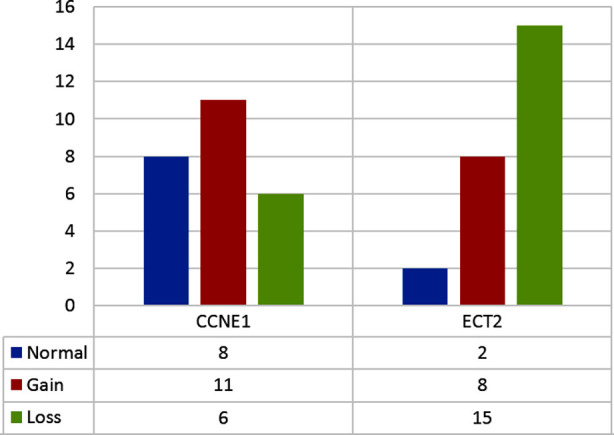
Frequency of *CCNE1* and *ECT2* CNVs in cases.

In HGSOC, CNG was observed in 11 cases (73.33%) while CNL was present in four cases (26.67%) of *CCNE1*. In LGSOC, both cases showed CNL in *CCNE1*. In *ECT2* in HGSOC the CNG was observed in seven cases (33.34%) while 14 (66.67%) cases showed CNL. In LGSOC CNG and CNL was observed in one case each, respectively. The highest frequency of copy number variations in *CCNE1* and *ECT2* gene were seen in FIGO Stage-III (Fig.2(b) and 2(c)).

**Fig.2(b):**
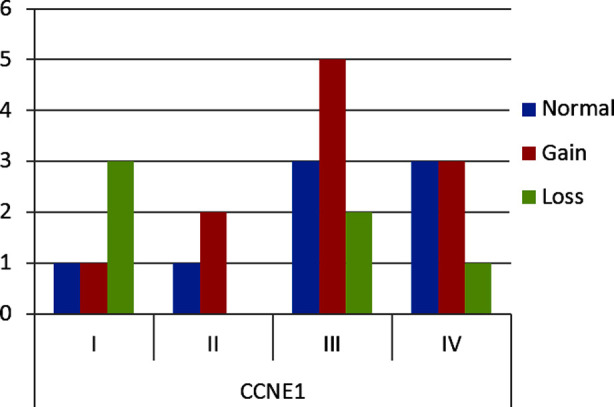
Frequency of *CCNE1* CNVs in FIGO stages.

**Fig.2(c):**
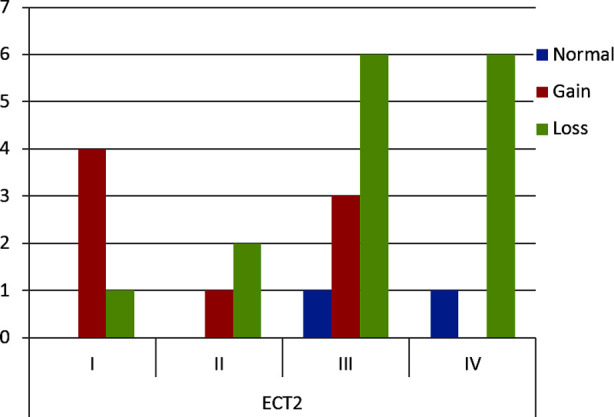
Frequency of *ECT2* CNVs in FIGO stages.

The multivariate analysis of chemotherapy response and CNVs showed that the risk of chemo-resistance was high with the CNVs of *CCNE1* while *ECT2* CNVs were related to chemo-sensitivity. However, p-values were statistically insignificant (Table-III).

**Table-III:** Multivariate analysis of chemotherapy response in *CCNE1* and *ECT2*CNVs.

Copy Number Variants	Chemotherapy Response		Multivariate Analysis		

	Sensitive n(%)	Resistant n(%)	OR Ratio	P value	95% CI
***CCNE1***					
Normal	7(35%)	1(20%)	Reference		
Gain	8(40%)	3(60%)	2.625	0.446	0.220-31.349
Loss	5(25%)	1(20%)	1.400	0.826	0.070-28.120
***ECT2***					
Normal	1(5%)	1(20%)	Reference		
Gain	7(35%)	1(20%)	0.143	0.272	0.004-4.612
Loss	12(60%)	3(60%)	0.250	0.373	0.012-5.262

Statistical test applied: Logistic regression, * P <0.05 is taken as statistically significant.

### Protein expression of CCNE1 and ECT2:

Analysis of pre-chemotherapy protein levels of *CCNE1* and *ECT2* in cases and controls showed that mean protein levels were high in cases than controls. This difference was statistically significant in CCNE1 (p-value <0.001) (Table-IV a). Pre and post chemotherapy mean protein levels of CCNE1 and ECT2 showed statistically significant difference (Table-IV b).

**Table-IV-a:** Difference between pre-chemotherapy mean protein levels of CCNE1 and ECT2 in cases and controls.

	Protein levels (Mean ± SEM)		Difference in levels (Mean ± SEM)	p-value

	Cases	Controls		
CCNE1(ng/L)	2189 ± 332.7	596.1 ± 41.77	1593 ± 374.7	0.001*
ECT2 (ng/L)	1540 ± 123.1	1468 ± 89.36	72.24 ± 148.6	0.802

Statistical test applied: Mann-Whitney U test,

* P <0.05 is taken as statistically significant.

**Table-IV-b:** Difference between Pre and post-chemotherapy mean protein levels in cases.

	Protein levels (Mean ± SEM)		Difference in levels (Mean ± SEM)	p-value

	Pre Chemotherapy	Post Chemotherapy		
CCNE1(ng/L)	2189 ± 332.7	1055.4 ± 836.2	1134 ± 294.4	0.001*
ECT2(ng/L)	1540 ± 123.1	875.2 ± 112.8	592.7 ± 36.8	0.001*

Statistical test Applied: Wilcoxon-signed Rank test,

* P <0.05 is taken as statistically significant.

## DISCUSSION

The present study included twenty-five SOC patients and twenty-five control subjects. The main focus of the study was to observe the role of CNVs and their protein expression in response to chemotherapy in relation with different clinico-pathological characteristics of SOC patients.

The age range of the patients in our study was from 18 to 70 years with a mean age of 47.16 ± 12.24 years. Similar mean age has been reported in ovarian cancer patients in other local studies.^4^,^17^ In SOC patients, age is an important prognostic factor. Older females have dismal prognosis compared to younger patients.^18^

The present study reported high percentage of HGSOC (92%) compared to LGSOC (8%). Nearly all national and international studies on SOC give a high percentage of HGSOC as compared to LGSOC.^6^,^17^-^19^

The present study reported 20% cases in FIGO Stage-I, 12% in Stage-II, 40% in Stage-III while 28% in Stage-IV. A 35 years cohort study from Scotland reported large number of cases (40%-50%) in Stage-III similar to the present study.^18^ Many national studies also report a high FIGO stage at presentation in OC patients.^4^,^17^,^20^

In the present study, mean difference between CA-125 levels (U/ml) at the time of diagnosis and after chemotherapy was statistically significant. The mean level of CA-125 (U/ml) in chemo-sensitive patients decreased significantly while it remained elevated in chemo-resistant patients after completion of chemotherapy.

Baseline CA-125 levels can be used as a significant prognostic and predictive factor in the management of HGSOC patients. They can help in determining disease free and overall survival in patients. The disease progression and regression can be predicted nearly in 90% of cases with variation in serum levels of CA-125.^7^,^21^

In the present study, 80% patients were chemo-sensitive while 20% showed resistance to chemotherapy. In a study by Zheng et al., 2021^5^ out of 60 OC patients 44 (73.33%) were chemo-sensitive while 16 (26.67%) showed chemo-resistance. It has been reported that HGSOC show a remarkable initial response to platinum therapy^4^,^6^,^17^. However, primary platinum-based chemo-resistance occurs in nearly 30% SOC patients, which is responsible for disease recurrence. Therefore, it becomes imperative to look for accurate and patient specific predictors for chemo-resistance and/or recurrence before starting treatment for these cancers.^16^

The present study identifies variations in copy numbers of CCNE1 as marker of chemo-resistance in ovarian cancers. Gorski et al.^10^ reported that the gain or amplification of *CCNE1* occurs in nearly 20% of all HGSOC and is associated with primary treatment resistance and decreased survival rate. Similarly, Wang et al^22^ in their meta-analysis concluded that *CCNE1* is a negative prognostic factor and is associated with poor overall survival in ovarian cancer patients. Buchynska et al.^15^ in their study on the role of *CCNE1* gene in endometrial carcinomas showed the gene amplification in 14.3% cases and a deranged protein expression in 65.6% of cases. However, the high grade serous variant of the tumor showed CNVs in 40% cases. We observed a similar pattern in our study population. Therapies that target this aspect may provide an opportunity to improve outcomes by tailoring the treatment regimen for patients with ***CCNE1***-amplified ovarian tumors.^10^

Dysregulation and increased expression of *ECT2* and its protein has been reported as a driver event in ovarian carcinogenesis and other malignancies.^23^,^24^ The present study reported an association of copy number gain and loss in *ECT2* with the chemo-sensitivity while protein expression after chemotherapy concurrently decreased with the CA125 levels. Contrary to this finding, most of the studies reported *ECT2* as a marker of bad prognosis in solid malignancies.^11^,^25^ Despite many molecular updates for ovarian carcinomas in the literature, still the overall survival of the patients is poor worldwide while it is graves in our part of the world. Copy number variations are lately been emphasized for their role in potential prognostication of SOC patients in different studies. This is the first ever study on the role of CNVs of two genes in serous ovarian carcinoma patients in Pakistan as this molecular aspect of the disease has not yet been explored even in nearby Asian countries. As tumour biology predicts the response to treatment and overall survival of the patients, the variations in the expression of CNVs observed in the present study delineates a potential link between molecular aberrations and poor response to standard chemotherapeutic regimens used for the management of our female population. This potential link however calls for further exploration and future large scale studies in our local population for better validation and generalizability.

### Limitation of the study:

This is a small scale study with limited numbers of patients and a six months follow-up. Further large scale studies with increased number of patients and long term follow- up are required to validate these findings.

## CONCLUSION

In conclusion, the copy number variations of *CCNE1* and *ECT2* and their protein expression are positively associated with the chemotherapy response in SOC patients and may serve as novel markers to predict the response to chemotherapy in local population.
